# Midfrontal theta reactivity to conflict and error are linked to externalizing and internalizing respectively

**DOI:** 10.1017/pen.2023.10

**Published:** 2024-04-25

**Authors:** Phoebe S.-H. Neo, Shabah M. Shadli, Neil McNaughton, Martin Sellbom

**Affiliations:** Department of Psychology, University of Otago, Otago, New Zealand

**Keywords:** Externalizing, Internalizing, Mental disorders, Personality, Theta rhythm

## Abstract

Dimensional psychopathology scores measure symptom severity; cutting across disorder categories. Their clinical utility is high given comorbidity, but their neural basis is unclear. We used scalp electroencephalography (EEG) to concurrently assess neural activity across internalizing and externalizing traits. “Theta rhythm” (4–7 Hz) spectral power at the frontal midline site Fz in specific goal conflict and action error phases within a trial of a Stop-Signal Task was extracted using process-specific contrasts. A final sample of 146 community participants (63 males, 83 females; mean age = 36; SD = 9; range = 18 – 56), oversampled for externalizing disorder (49% diagnosed with a DSM-5 externalizing disorder), also supplied psychopathology and personality data. We used the Minnesota Multiphasic Personality Inventory−3 (MMPI-3) to measure symptoms and traits of psychopathology. An MMPI-3 measure of the higher-order internalizing psychopathology spectrum was positively correlated with action error theta. An MMPI-3 measure of the higher-order spectrum of externalizing psychopathology was negatively correlated with goal-conflict theta. We showed that goal-conflict and error theta activity are higher-order processes that index psychopathology severity. The associations extend into the nominally healthy range, and so reflect theta-related factors that apply to the general population as well as patients with sub-threshold diagnoses.

The current Diagnostic and Statistical Manual of Mental Disorders (DSM-5) lists ∼300 categories of psychiatric disorders (American Psychiatric Association, 2013). While clinically useful, its nosology does not account for comorbidity and transdiagnostic features (Cuthbert, 2022; Krueger et al., 2018). Hierarchically-structured dimensional models of psychopathology are increasingly seen as important targets for the study of neural mechanisms that are common across psychiatric disorders (Kotov et al., 2018; Michelini et al., 2021).

Analysis of co-occurring psychiatric diagnoses (Kessler et al., 2011; Krueger, 1999) extracts two higher-order spectra of covariance cutting across DSM-5 categories (often with a third spectrum of “thought disorder,” Kotov et al., 2017). “*Internalizing”* spans mood and anxiety disorders (Kessler et al., 2011; Krueger, 1999) such as major depression, generalized anxiety, social anxiety, and panic disorder. Internalizing correlates with a broad range of clinical and personality trait dimensions that reflect dysfunctional, high negative, and/or low positive, affect (Naragon-Gainey et al., 2018; Sellbom et al., 2008). “*Externalizing”* spans disorders of disinhibition and socially deviant behavior (Krueger et al., 2021) such as attention deficit hyperactivity, antisocial personality, and alcohol and substance use. Externalizing correlates with a broad range of clinical and trait dimensions that reflect poor impulse control, and social and behavioral antagonism (Krueger et al., 2021; Krueger et al., 2007).

Currently, neural studies using dimensional measures of the higher-order domains of internalizing and externalizing are limited (Krueger & Markon, 2011; Michelini et al., 2021). Existing work has largely examined categorical differences between those with a disorder and healthy controls (e.g., McLoughlin et al., 2021; Pasion & Barbosa, 2019). A recent review of the extant literature identified midfrontal theta activity (4–7 Hz) as providing important indices of cognitive vulnerabilities across psychiatric disorders that form not only part of the internalizing (anxiety, OCD) but also externalizing (ADHD and substance abuse) spectrum (McLoughlin et al., 2021). It should be noted that the theta indices reviewed (McLoughlin et al., 2021) spanned different study tasks and conditions, and also included non-oscillatory evoked potentials (e.g., the N2 and error-related-negativity, ERN) that have been associated with phase resetting of ongoing rhythmic theta activity (Cavanagh et al., 2012; Yeung et al., 2007). So, “midfrontal theta” measurements are functionally heterogeneous, and different mechanisms (Cohen, 2014) likely mediate the theta associations of different forms of psychopathology.

Theta rhythms increase in amplitude with signals of novelty (Harper et al., 2017), negative feedback (Ellis et al., 2018), conflict (Cohen & Donner, 2013; Neo et al., 2011; Pinner & Cavanagh, 2017) and error (Cohen, 2011; Pasion & Barbosa, 2019; Yeung et al., 2004; Zavala et al., 2016). These different activations may reflect a fundamental control process that is generally required when internal or external goals are changing, and require attention and monitoring (Cohen, 2014; McLoughlin et al., 2021). Of particular relevance here, it has been shown that midfrontal theta activity is hyper-reactive (heightened attention) in internalizing (Cavanagh & Shackman, 2015; Hajcak et al., 2019); and hypo-reactive (poor monitoring) in externalizing (Burwell et al., 2014; Hall et al., 2007; Yoon et al., 2013).

This opposite relation of midfrontal theta activity to internalizing versus externalizing has not yet been assessed within the same sample. Studies have been restricted to either internalizing or externalizing psychopathology. Does a single form of theta activation by a single event exert opposite impacts on internalizing and externalizing; and, if so, how? Theoretically, collateral input to different areas would make this possible. For example, increases in dopamine can have opposite effects on reward and punishment processing (Jean-Richard-Dit-Bressel et al., 2018). Also, if this is the case, do theta reactivities to different events show similar opposing links with internalizing and externalizing?

To address these questions, in the current study, we parsed theta activity in the Stop-Signal Task (SST, Logan et al., 1984) with different statistical contrasts applied to distinct periods within a trial to extract midfrontal theta activity specific to “*conflict,”* and *“error.” We then* tested both the *conflict* and the *error* associations with dimensional measures of both internalizing and externalizing.

In the SST, on GO trials participants make a mouse click as quickly as they can to a go-signal. Occasionally, STOP trials occur when a stop signal occurs after the go-signal; and participants should inhibit their mouse click. Both *conflict* and *error* are assessed by first subtracting power differences between STOP and GO trials and then applying different contrasts applied at different time points.

For *conflict,* measurements are made locked to the timing of the stop signal in STOP trials. The stop signal is presented with short, medium, and long delays, which deliver *low*, **high** (stop and go are similar), and *low* conflict conditions, respectively. Contrasting *high* with the average of *low* conflict generates an index of theta activity specific to *conflict* described in the neuropsychological theory of the Behavioral Inhibition System (BIS) (Gray & McNaughton, 2000). Here, the *conflict* is a result of the competing goals of quick responding and its inhibition, postulated to peak in trials with medium stop-signal delays. SST studies have shown such *conflict* activity at the midfrontal site Fz (McNaughton et al., 2013; Neo et al., 2011).

For *error*, measurements are made locked to the timing of the mouse click. In the SST, an error occurs when a participant fails to stop clicking on a STOP trial. So, on STOP trials, a click represents a mistake, in contrast to a click in GO trials. To extract processing specific to error, we contrasted failed STOP with successful GO trials, ignoring stop-signal delay. The majority of the EEG studies on error processing have focused on the ERN (Pasion & Barbosa, 2019). As mentioned earlier, the ERN is a response-locked potential, but is also associated with midfrontal theta rhythmic activity (Cavanagh et al., 2012; Taylor et al., 2007; Yeung et al., 2007).

Based on the literature just reviewed, which suggests hyper- and hypo-midfrontal reactivity in a range of internalizing and externalizing disorders, we hypothesized that both *conflict* and *error* theta activity at the midfrontal site Fz, would be positively correlated with the higher-order domain measure of internalizing; and negatively correlated with externalizing. Given the positive correlation between internalizing and externalizing (Krueger & Markon, 2006), we also predicted that the hypothesized relationships would be mediated by non-shared factors, that is, via theta variances specific to internalizing and externalizing respectively.

## 1. Methods and materials

### 1.1. Participants

The data were extracted from an archival database collected as part of a larger study on externalizing disorders and have not been previously published. Participants were recruited from the community with Facebook advertisements and flyers designed to target a sample over-weighted towards externalizing psychopathology. In two separate advertisements, we advertised for 1) individuals to take part in a brain and personality study; and 2) for individuals with drug, alcohol, or anger management problems – or with ADHD or impulsivity. After exclusion (see section on exclusion below), the final sample size consisted of 146 participants (63 males, 83 females; mean age = 36; SD = 9; range = 18 – 56). Structured clinical interviews (First et al., 2015, 2016) found 49% of the final sample met criteria for at least one DSM-5 externalizing diagnosis: ADHD (any type, 27%); history of conduct disorder (23%); antisocial personality disorder (21%); alcohol use disorder (18%); and cannabis use disorder (12%). These rates are substantially higher than a typical community sample (Kessler et al., 2011), consistent with overweighting towards externalizing problems during recruitment. The remaining half of the sample included healthy controls for externalizing disorders. Ethical approval was provided by the University of Otago Ethics Committee (Health), Approval number: H16/031.

### 1.2. Personality and psychopathology measures

The Minnesota Multiphasic Personality Inventory−3 (MMPI-3) (Ben-Porath & Tellegen, 2020) measures maladaptive symptoms, behaviors, and personality traits in accordance with contemporary theories of psychopathology and personality (Sellbom et al., 2021); and is aligned with modern dimensional models of psychopathology (Simms et al., 2021). It is designed for clinical assessment and has representative general community norms. It includes 335 true/false items that aggregate onto 10 validity scales and 42 substantive content scales that consist of Higher-Order (HO) scales; Restructured Clinical (RC) scales; Specific Problems (SP) scales; and the PSY-5 scales. The HO, RC and SP scales are hierarchically organized with item overlap of scales across (but not within) each level. The HO scales of Emotional/Internalizing Dysfunction (EID) and Behavioral/Externalizing Dysfunction (BXD) represent the highest order and therefore, most generalized measurements of internalizing and externalizing syndromes. EID and BXD form the primary measures of internalizing and externalizing in our hypotheses testing.

To facilitate comparisons with existing work on the associations between personality and psychopathology, we also used the Behavioral Inhibition (BIS) and Behavioral Activation System (BAS) scales from Carver and White’s (Carver & White, 1994) BIS/BAS measure. The BIS and BAS scales are widely used as self-report measures of neurobiological systems that mediate personality traits in the Reinforcement Sensitivity Theory (RST) (Corr & McNaughton, 2008; Krupić et al., 2016). Consistent with the dimensional models of psychopathology, in the RST, personality traits, when manifested at extreme levels, are significant contributors to psychopathology. Neural BIS contributes to internalizing; and neural BAS contributes to externalizing (Corr & McNaughton, 2014). The BIS/BAS scales are administered in a single questionnaire that consists of 20 items where participants rate on a 1–4 scale the extent that they agree with each statement/item. The BAS consists of three sub-scales: Drive, Fun Seeking and Reward responsiveness. To reduce multiple testing, we used the total score of the sub-scales here. Although the MMPI-3 inventory also includes scales of abnormal personality, that is, the PSY-5 scales (Harkness et al., 2012), like the other scales in the MMPI-3 inventory, they were excluded in our analyses due to overlapping items with EID and BXD.

### 1.3. The stop-signal task (SST)

**Figure 1.**
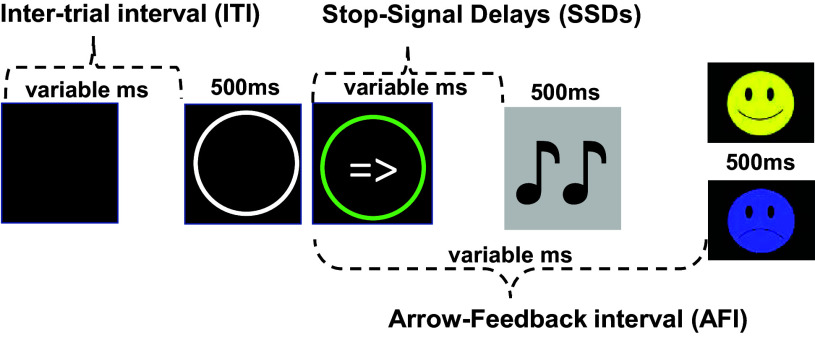
Sequence of events in a trial. Onset of the tone from the time of the arrow presentation (SSDs) in a Stop trial are variable. A Go trial follows the same event sequence but without the onset of the tone. A smiley/frowny face is presented for a successful/unsuccessful withholding of a mouse click in a Stop trial. In a Go trial, a smiley/frowny face is presented for correct/incorrect responses. ms: milliseconds. ITI: 500 ms to 4000 ms; SSDs: see Table 1; A FI: Go Correct = 1000 ms; Stop Fail = 1500 ms; Stop Correct = 1700 ms.

The SST was originally designed to assess motor inhibition (Logan et al., 1984). Here, we used an SST version, as described in Shadli et al. (2015), modified to optimally assess “*conflict.”* On a GO trial, participants make a left/right mouse click as fast as possible when they see a left/right arrow. As shown in Fig. 1, on a STOP trial, a tone sounds after the onset of the arrow, signaling the task requirement to withhold the mouse click. The tone occurs only occasionally (1 STOP trial counterbalanced amongst 3 GO trials). The Stop-Signal Delay (SSD) determines when the tone is presented from the onset of the arrow. As SSD increases, the chances of failing to inhibit the motor response also increase. STOP trials with short, medium, and long delays were distributed across the task using separate “staircases”. Short delay and long delay staircases adjusted the SSDs depending on prior reaction times averaged over recent GO trials. The medium delay staircase SSD was adjusted up or down by 30 ms after correct or failed stopping, respectively (intended to track 50% stopping). There were 99 STOP trials and 296 GO trials in total spread over three blocks with rest breaks in between. Prior to the test trials, participants completed 30 practice GO trials with no STOP trials.

### 1.4. EEG acquisition and processing

EEG recording was referenced to CPz and sampled at 512 Hz. EEG was recorded from FP1, FPz, FP2, F7, F3, Fz, F4, F8, T7, T8 C3, Cz, C4, P7, P3, Pz, P4, P8, M1 and M2 with an Advanced Neuro Technology (ANT) amplifier and ANT caps with AgCl electrodes. Impedances were tested with ANT software (eego) and reduced to below 10 KΩ. Data from 30 participants were sampled at 256 Hz. Given that the analysis was of power *averaged* over 4–7 Hz, and since this is well below 256 Hz, these were analyzed as sampled. The MATLAB 2019a plugin, EEGlab (version 2019_0), was used to process the data. The EEG of all 18 recording sensors was re-referenced to the mastoid sensors (M1 and M2); filtered from 1 to 36 Hz using the function (pop_ eegfiltnew); and segmented from the start of the first to the end of the last test trial. We used the runamica15() function to run Independent Component Analysis to identify eye blink and movement components. The eye components were removed automatically using the pop_icflag() function. A 90% similarity threshold was used, that is, only components with > 90% similarity to eye components were removed. Next, two 1 5 s EEG segments centered, respectively, at the onset of the Stop-Signal and mouse click were separately extracted from each trial. Any segment with EEG > ±70 µV detected in any of the sensors was excluded from analyses using the function (pop_eegthresh). Two consecutive 1 s Hanning windows, overlapping by 0·5 s, were applied to each 1 5 s segment, delivering two 1 s epochs. The Hanning window is a cosine function that reduces the weighting of the edge data within an epoch. (Note that the overlapping and windowing procedures delivered, in the next step, spectral activity centered on the 0·5 s after the onset of the Stop-signal and mouse click respectively.) The fft() function was used to extract spectral amplitude from each 1 s epoch; squared to derive spectral power; and log transformed to normalize the power distribution. The normalized spectral power for 4–7 Hz was then averaged to yield a single value to index theta activity at each period of interest. Below, we described further steps for deriving the “*conflict”* and “*error”* contrasts.

GO trial activity was used as a baseline to control for GO response-related theta. In the SST, all STOP trials began in the same way as a GO trial until the onset of the stop signal, which initiated processing of STOP, superimposed on existing GO processes. So, the STOP-GO difference extracts STOP-specific theta activity removing other general and GO-related processes. For both *conflict* and *error,* theta power in the GO trial preceding a STOP trial was subtracted from that STOP trial (power from the following GO trial was subtracted if the preceding trial was also a STOP trial). As the sequence of STOP and GO trials was pre-determined and fixed across participants, the GO trials used for the computation of STOP-GO activity was pre-identified. Post-experiment, an event mark was inserted into these GO trials according to the SSDs of the STOP trial it was paired with. For example, if the SSD for the STOP trial was 20 ms, a unique event mark was inserted 20 ms from the onset of the arrow in the paired GO trial. Spectral power was then extracted from this “Stop-Signal” event mark as per the procedures for STOP trials.

The *conflict* contrast was computed by averaging these STOP-GO trial differences for early and late SSDs and subtracting this average from the average of trials with medium SSDs. The residual theta power (centered on 500 ms from the onset of the stop signal) indexed inhibition-related reactivity specific to *conflict,* which should peak in the medium SSD trials because response inhibition was relatively easy or hard in the early- and late-delay trials, respectively, while stopping and going were in balanced conflict in the medium case. As we were interested in trait-like neural responses that are relatively stable over time, we extracted a minimum of 5 trials per SSD trial type from each of the 3 blocks of SST trials. Participants with too few trials from any of the blocks (due to EEG noise) were excluded from the analyses.

The *error* contrast was computed centered on the 500 ms from the onset of the mouse click (in both STOP and GO trials); and only STOP trials with mouse clicks (failed inhibition) were averaged as were GO trials with mouse clicks. Thus, clicking was consistent and trials only differed in terms of whether the response was incorrect (STOP trial) or correct (GO trial). Participants with fewer than 15 trials in the averages were excluded.

### 1.5. Participant exclusion

Out of 218 participants, 47 participants were excluded due to excessive EEG artifacts. Compared to our previous work that did not focus on externalizing, a relatively larger percentage of participants were excluded. More of our participants fidgeted throughout the task, generating EEG artifacts, likely due to externalizing (e.g., ADHD) symptoms. This interacted with the requirement here for a minimum number of trials from each block of SST trials for analysis, leading to a larger number of exclusions. A further 25 participants were excluded due to inconsistent or deviant responding, detected with the MMPI-3 validity scales (Ben-Porath & Tellegen, 2020).

### 1.6. General procedures

Participants took part in the experiment as part of a larger study; they completed a battery of questionnaires that included the ones reported here as sub-sets. While they completed the questionnaires, the experimenter prepared the participants for EEG recording by applying electro-gel and reducing the impedance of the recording sensors attached to the EEG cap. The participants were then tested (∼25 min) on the stop-signal task (Shadli et al., 2015), followed by the gold bar/lemon task (Neo et al., 2021) (∼30 min; data not reported here). The participants were then administered the structured clinical interviews. The experimental session was conducted by a trained research assistant under supervision by a registered clinical psychologist. At the end of the experiment, participants were paid $50 in petrol or supermarket vouchers, and cash winnings ($6) from the gold bar/lemon task. The whole experimental session lasted 3 5 – 4 h.

### 1.7. Data analysis

To facilitate comparison with published SST studies, we computed the descriptive statistics for the behavioral measures commonly reported in the SST (Table 1). Our data came from an archival database and so participants with internalizing problems were not specifically targeted during recruitment. However, externalizing and internalizing problems are moderately correlated (Krueger & Markon, 2006). To ascertain that our sample, recruited for overweighting in externalizing symptoms, showed the appropriate range in scores for both externalizing and internalizing, we calculated the descriptive statistics of the self-report measures (Table 2).

To assess if midfrontal (Fz) *conflict and error* were associated positively/negatively with dimensional measures of internalizing/externalizing and personality traits implicated in psychopathology, we calculated the Pearson product-moment correlations for each of our theta measures with EID/BXD and BIS/BAS scales respectively (see Table 2). To index midfrontal activity, we used Fz and the mean of 4–7 Hz theta activity. These choices, including the spectral power extraction methods, were made so as to replicate the methods used in previous SST work (McNaughton et al., 2013; Neo et al., 2011; Shadli et al., 2015) on goal conflict, defined by the theoretical BIS (Gray & McNaughton, 2000). Although other sensors, frequencies and spectral power extraction methods could potentially generate stronger results, keeping these unchanged here facilitates comparison with the previous work. More importantly, the usual way of identifying optimal sensors and frequencies with a spatial map of mean spectral power across electrodes was problematic for our current purpose. Our aim was to identify neural activity that varies with individual differences in psychopathology dimensions. Large mean activity in sensors/frequencies is not equivalent to variability across individuals. Therefore, we anchored our *methods* of analysis to previous SST versions set within the theoretical framework of BIS which is compatible with the dimensional perspective of psychopathology (for details on how BIS fits in with the dimensional perspective, see Corr & McNaughton, 2008). Our hypotheses on the relationships between theta, internalizing, and externalizing are also anchored to the wider literature on midfrontal theta activity and its contributions to different form of psychopathology.

To assess if midfrontal theta effects were distributed across other sensors, the following steps were repeated for both *conflict* and *error* to create an index of distributed theta activity. Firstly, 4–7 Hz STOP-GO theta differences of all 18 active electrodes were entered into a Principal Component Analysis (PCA) to extract a single major component. Next, the eigenvalues of the full model were compared to outputs from a parallel analysis to help us determine the inherent structure of the data, that is, the optimal number of components to extract. Regardless of the results of the parallel analysis, individual regression-weighted scores of the first component in the solution were used to index variation in theta activity distributed across the 18 electrodes. (For a solution with more than 1 factor, the oblique Promax rotation to simple structure was used.) We then calculated the *Pearson* correlations between the individual factor scores and the self-report measurements listed in our hypotheses (see component 1 results in Table 2). The component loadings of each of the 18 electrodes for *conflict* and *error* are shown in Fig. 3.

To assess shared versus unique variances, we entered EID and BXD; and in a separate regression model, BIS and BAS; as predictors of midfrontal theta activity to extract the shared and unique variances. To assess if age could account for any of the results above, we also extracted *Pearson* correlations between age and each of our dimensional measures of internalizing and externalizing.

To facilitate comparisons with previous work focused on group averages, we plotted the spectral power, averaged across all participants, for *conflict* and *error* (see Fig. 3). However, note that it was not appropriate to test the group means for statistical differences as the means were computed from different contrasts reflecting fundamentally different cognitive processes. Our aim here was to help the reader, used to analyses of group averages, gain qualitative insights into the distribution of individual theta contrasts across the two conditions. Conversely, those used to individual self-report data would be interested in the reliability of the measures (Hajcak et al., 2017). These are difficult to calculate in this case (see Clayson et al., 2021, on reliability of difference scores) as they involve complex contrasts (e.g., linear x quadratic over a set of 6 trials). Assessing our type of measure like a standard single, conventional, score has additional problems since: 1) the score varies systematically across trials (Shadli et al., 2021) – which would add to the nominal error of a conventional reliability score; and, 2) is not meant to be test-retest reliable (Shadli et al., 2020).

## 2. Results

The common behavioral measures published in SST studies are shown in Table 1. The current sample showed slower reaction times, and the percentage of successful STOP trials was also lower across all types of trials compared to previous work (Aron & Poldrack, 2006; Shadli et al., 2015). Notably, seven participants failed to inhibit their responses throughout the task. That explains the zero values in Table 1 (see min. values for successful inhibition, row 1–3). With over 80% correct responses in GO trials, these participants did not appear to be ignoring the task requirements. Also, the computation of our contrasts was not dependent on correctly inhibited trials. So, even though these participants had zero trials available for correctly inhibited SST trials, they were included in our EEG analyses.

**Table 1.** Descriptive values in the current study of behavioral measures commonly reported in the SST

	Min	Max	Mean	SD
Inhibit SSD early (%)	0·00	100·00	50·77	28·36
Inhibit SSD medium (%)	0·00	68·75	32·57	17·52
Inhibit SSD late (%)	0·00	50·00	9·37	10·17
SSD early (ms)	71·09	154·64	102·66	17·19
SSD medium (ms)	124·79	387·69	194·33	54·10
SSD late (ms)	286·35	619·52	407·27	68·92
GO reaction time (ms)	349·50	788·00	502·97	93·02
SS reaction time (ms)	170·83	460·42	308·64	63·93
Correct GO (%)	86·20	100·00	96·82	3·23
Failed STOP reaction time (ms)	328·00	774·00	473·58	79·56
Failed STOP SSD (ms)	154·79	520·27	263·03	74·15

Row 1–3: percentage of successfully inhibited mouse clicks for early, medium and late SSD trials respectively; row 4–6: length of SSD for each trial type; row 7: reaction times in GO trials; row 8: Stop-Signal Reaction Times (mean SSD for medium trials subtracted from median GO reaction times); row 9: percentage of correct responses in GO trials; row 10–11: reaction times and length of SSD for failed STOP trials.

The descriptive statistics of the self-report measures are shown in Table 2. It shows that our sample showed average to above average internalizing scores (see EID) and with even greater variability than is typical of a community sample (Ben-Porath & Tellegen, 2020). It also shows that, as expected given our recruitment strategy, there is a high variability in externalizing scores (see BXD). Table 2 also shows that *error* theta, whether indexed by Fz or component 1, was positively correlated with EID. Although error theta at Fz showed a positive correlation with BIS, this was not significant after Bonferroni correction for multiple testing. (Given that each unique theta index was tested 4 times, we used a correction factor of 4). Both Fz and component 1 *conflict* theta were negatively correlated with BXD. Similar negative correlations were observed between *conflict* theta and BAS. The conflict:error correlation for Fz was r(144)  = +0·145, NS, and their relative involvement in BXD and EID is discussed below (see also Fig. 5).

**Table 2.** (left)Trait scores and (right) correlations for STOP-GO theta (4–7 Hz average) power against trait measures at midline (Fz) and component 1 at two different time points in the SST (conflict, error)

	Trait Scores				Conflict		Error	
	Min	Max	Mean	SD	Fz	Com 1	Fz	Com 1
Emotional/Internalizing Dysfunction	31·90	88·97	55·73	11·96	−0·10	−0·10	0·19**	0·20**
Behavioral Inhibition System	1·43	4·00	3·07	0·56	0·11	0·09	0·14*	0·12
Behavioral/Externalizing Dysfunction	32·59	87·08	57·19	14·56	−0·19**	−0·18**	0·07	0·08
Behavioral Activation System	2·00	3·92	3·14	0·42	−0·21**	−0.18**	0·08	0·02

* Correlation is significant at the 0·05 level (1-tailed).

** Correlation is significant at the 0·01 level (1-tailed).

**Figure 2.**
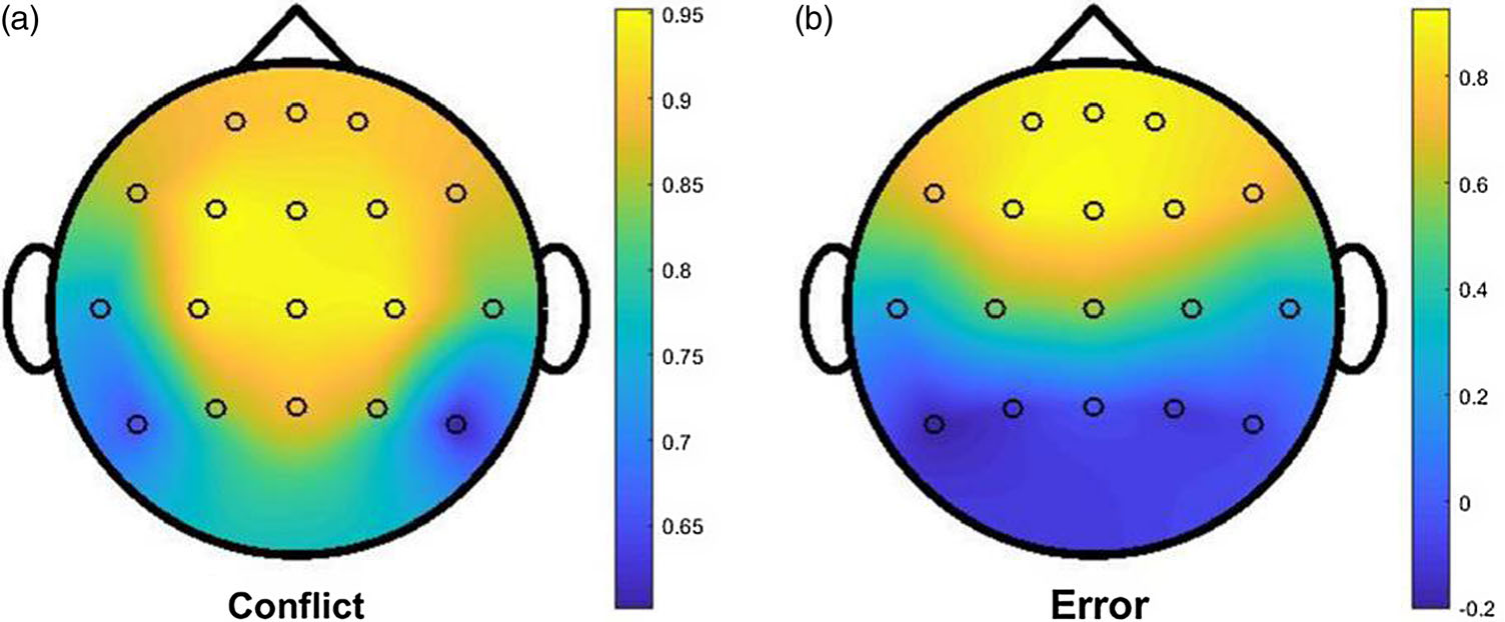
Relative contributions of STOP-GO theta activity from each electrode to component 1 at two different time points in the SST (conflict, error). (a) Electrode loadings for conflict from its component matrix. (b) Electrode loadings for error from its pattern matrix. Warmer colors indicate relatively higher loadings. Electrode positions, from left to right: 1^st^ row, Fp1, Fpz, Fp2; 2^nd^ row, F7, F3, Fz, F4, F8; 3^rd^ row, T7, C3, Cz, C4, T8; 4^th^ row, P7, P3, Pz, P4, P8.

The head maps in Fig. 2 show how theta activity from each of the 18 electrodes loaded onto component 1 in *conflict* and *error* respectively. Results from the parallel analyses suggest that the data structure for *conflict* and *error* consisted of one and two components respectively. Therefore, the contribution of each electrode to component 1 for *conflict* (Fig. 2a) was extracted from a component matrix (unrotated solution) and that for *error* (Fig. 2b) was extracted from a pattern matrix (rotated solution). Figure 2a shows that component 1 for *conflict* has the highest loadings in a central frontal cluster of electrodes consisting of F3, Fz, F4, C3, Cz and C4. Figure 2b shows that component 1 for *error* has the highest loadings in a frontal cluster of electrodes consisting of Fp1, Fpz, Fp2, F3, Fz, F4.

**Figure 3.**
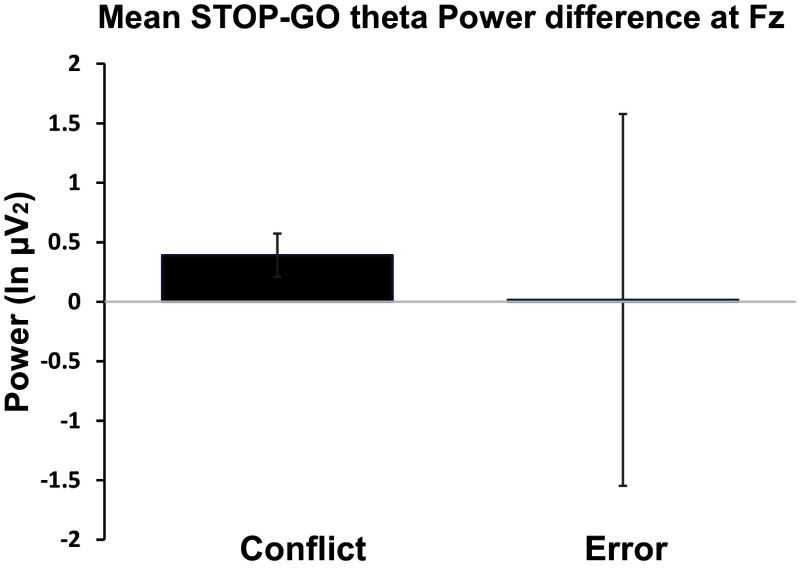
Mean spectral power of STOP-GO theta activity (4–7 Hz) *at two different time points in the SST and using two different contrasts (conflict, error).*

Consistent with previous work (Krueger & Markon, 2006), EID and BXD were positively correlated *r*(146) = 0·44, *p* < 0·001. Age did not show any consistent pattern of correlations with either internalizing or externalizing measures in the current sample. Our regression analyses showed that the significant correlations (after correction for multiple testing) observed in our study (see Table 2) were due to unique variances (see Table 3).

**Table 3.** R square variances of regressions with trait measures as predictors of STOP-GO theta (4–7 Hz average) power at midline (Fz) and component 1 at two different time points in the SST (conflict, error). A. Regressions with EID and BXD as predictors. B. Regressions with BIS and BAS as predictors

	Conflict				Error			
	Fz		Component 1		Fz		Component 1	
	Model	Unique	Model	Unique	Model	Unique	Model	Unique
**A** Emotional/Internalizing Dysfunction	0·04*	0·00	0·03	0·00	0·04	0·03*	0·04	0·03*
Behavioral/Externalizing Dysfunction		0·03*		0·03		0·00		0·00
**B** Behavioral Inhibition System	0·06*	0·01	0·04*	0·01				
Behavioral Activation System		0·04*		0·03*				

* Variance of regression model or unique contribution is significant at the 0·05 level (1-tailed).

**Figure 4.**
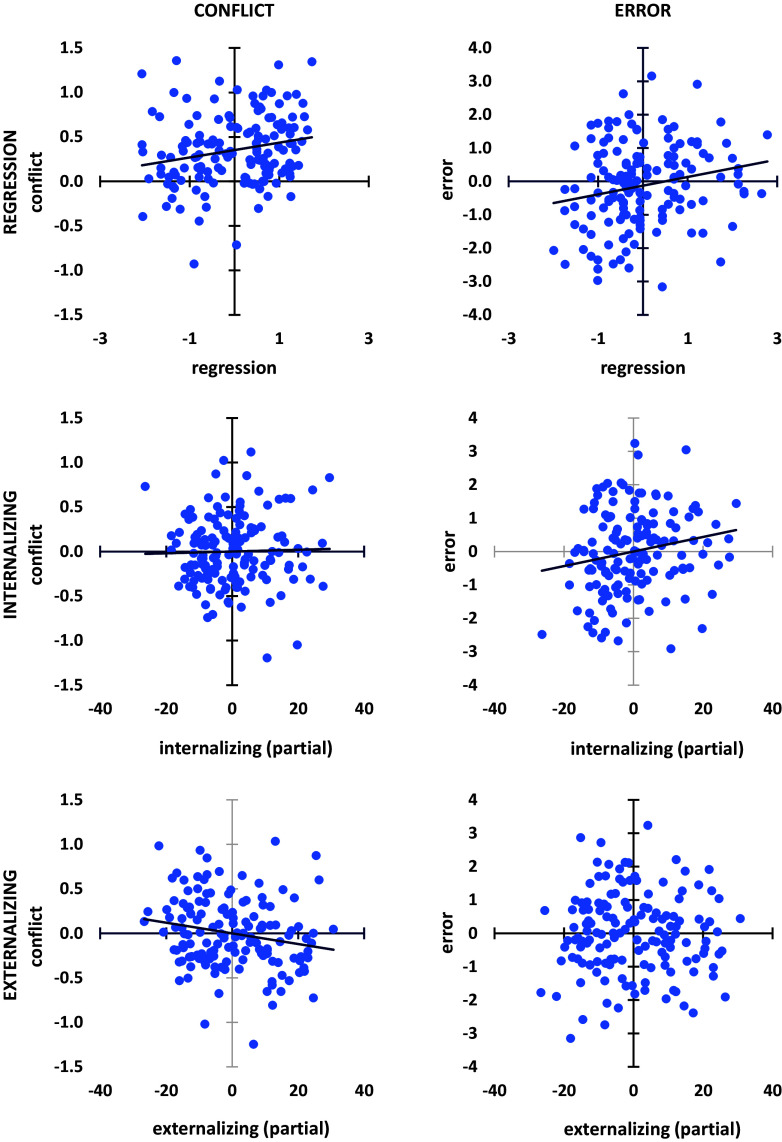
Scatter plots (with regression lines) for overall regression model and internalizing and externalizing partial components for conflict and error. Note that the partial correlation for error with internalizing is positive, while the partial correlation for conflict with externalizing is negative.

In Fig. 3, we also show the means of *conflict* and *error* at the midfrontal site Fz. The mean spectral theta power measures of *conflict* and *error* showed distinct characteristics. *Conflict* activity was of the same order of magnitude as reported previously (Shadli et al., 2021, 2015) with a positive mean (µ = 0·17 log_10_ μV^2^). *Error* activity was close to zero (µ = 0·007 log10 μV^2^). Notably, standard error for *error* was much larger compared to *conflict* (SEM = 0·68). As the contrast used in the two cases is different, statistical comparison of variances is not appropriate. As mentioned in our data analysis section, in contrast to analyses of group averages, our aim was to identify neural activity that varies with individual differences in psychopathology dimensions. So, the interpretation of a small mean in group analyses as a lack of significant activity is less relevant here. These differences in means and variance are also clear in the plots of the overall regression and partial internalizing and externalizing components shown for conflict and error in Fig. 4.

**Figure 5.**
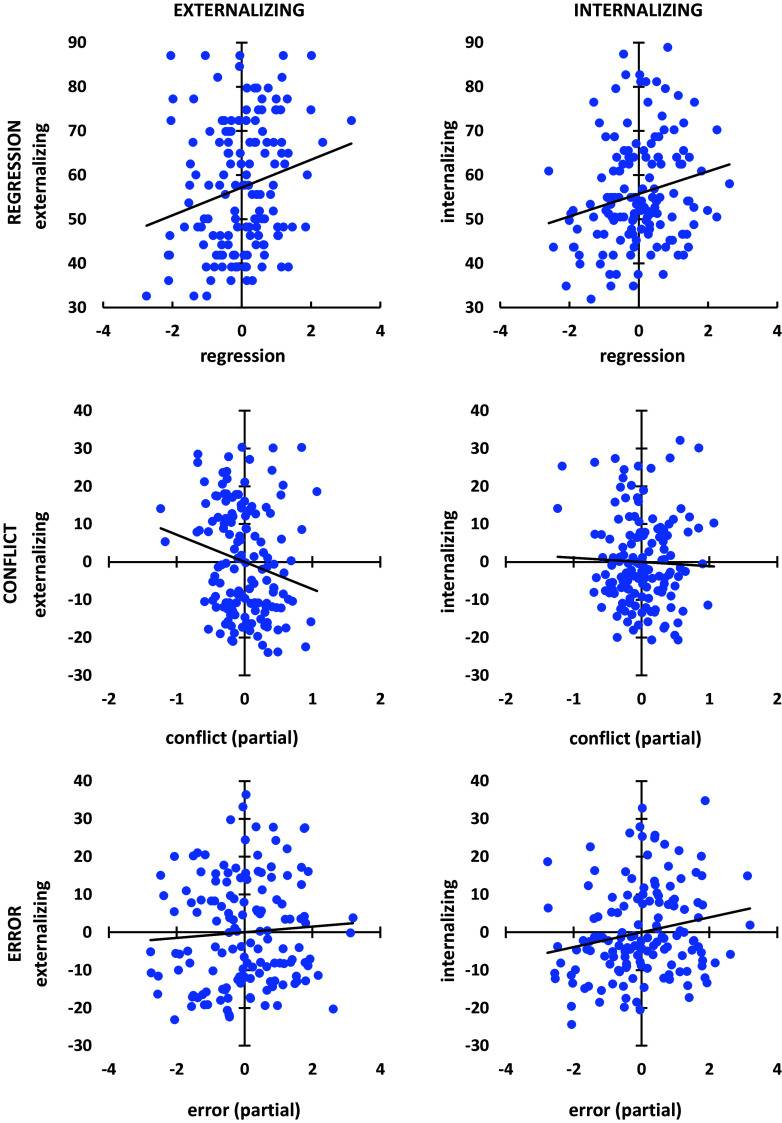
Scatter plots (with regression lines) for overall regression and conflict and error partial components for externalizing and internalizing. Note that the partial correlation for conflict with externalizing is negative while the partial correlation for error with internalizing is positive.

To further explore the relation between conflict and error, we entered them as predictors in separate regressions for externalizing and internalizing. In both cases the overall regression was significant (p = 0·033, p = 0·036, respectively). For externalizing, this was primarily due to a unique negative contribution from conflict (part correlation = −0·195) with an apparent slight positive contribution from error (part correlation = 0·064. For internalizing, in contrast, this was primarily due to a unique positive contribution from error (part correlation = 0·202) with an apparent slight negative contribution from conflict (part correlation = −0·036). Scatter plots for these relationships are shown in Fig. 5.

## 3. Discussion

Midfrontal theta activity reflecting action errors was positively associated with internalizing psychopathology. Its association with the BIS personality scale was also positive but the relationship did not survive correction for multiple testing. Midfrontal theta activity reflecting *conflict* was negatively associated with both externalizing psychopathology and the BAS personality scale. When STOP-GO theta activity distributed across the frontal, central and posterior regions of the scalp were reduced to extract a major theta component, a central frontal cluster of sites loaded highly on the component for *conflict*; and a frontal cluster loaded highly on the component for error. Both components showed the same associations as their respective midfrontal theta at Fz displayed with the self-report measures of psychopathology and personality.

These results are consistent with the idea that theta activity indexes important psychiatric vulnerabilities (Cavanagh & Shackman, 2015; Corr & McNaughton, 2014; Gray & McNaughton, 2000; McLoughlin et al., 2021). More specifically, given that EID and BXD operationalize higher-order domains that capture common variance cutting across traditional mental disorders, our results suggest that *conflict* and *error* theta activities reflect higher-order mechanisms that underly psychopathology. These heterogeneous theta activities appear to share the capacity to support higher-order cognitive functioning that is generally required when internal or external goals are changing or in conflict, and require attention and monitoring (Cohen, 2014; Gray & McNaughton, 2000; McLoughlin et al., 2021). Additionally, both *conflict* and *error* theta activity were quantitatively related to the symptoms, behaviors and traits of psychopathology, extending into the healthy range. This supports the dimensional model of psychopathology (Cuthbert, 2022; Kotov et al., 2018; Krueger et al., 2018; Michelini et al., 2021), which suggests that there are no clear breaks between health and disorder.

We did not find support for our prediction that a single theta process would contribute to both the internalizing and externalizing domains. This finding is theoretically possible, since the same process could interact (in opposite directions) with a distinct set of co-factors in the development of internalizing and externalizing, respectively; or result as a symptom.

For example, *conflict* activity in anxiety biased samples has been linked to facets of internalizing (Shadli et al., 2021). But, here, each of our theta measures was correlated (via unique variances) with either internalizing or externalizing but not both. Conversely, each of internalizing was correlated with either error or conflict, but not both. This finding could be a result of sampling biases. Unlike the previous study by Shadli et al. (2021) that targeted patients with anxiety symptoms, the current study oversampled for externalizing behaviors and traits. Our participants showed slower Go trial reaction times and lower percentages of successful inhibition than previously (Aron & Poldrack, 2006; Shadli et al., 2015). This result could be due to poor attention span, since half of our participants had diagnosable externalizing problems, with almost a third being diagnosed with ADHD. Although internalizing scores are high in our sample, these could be a consequence of externalizing problems and therefore have inherently different biological origins than a sample with internalizing symptoms as the primary signs of psychopathology. This highlights the importance of multi-modal assessments in psychopathology in understanding their causes. To this end, midfrontal theta activity appears to be a good candidate for indexing psychiatric vulnerabilities.

We must also comment on the lack of relation between externalizing and error theta. As noted in the introduction, midfrontal theta activity has been shown to be less reactive (linked to poor monitoring) in externalizing (Burwell et al., 2014; Hall et al., 2007; Yoon et al., 2013). Hall et al. used a flanker task with students with extreme scores on the Externalizing Spectrum Inventory, the other two studies used oddball tasks with groups with “lifetime” externalizing diagnoses. It is most likely that our lack of similar findings reflects the more motoric nature of our task; but, in any case, all three of these studies obtained their clearest results at fronto-central and posterior sites with small or null results in the region of our frontal (Fp1, Fpz, Fp2, F3, Fz, F4) cluster.

In particular, we show here that reaction to response errors (*error*); and insufficient sensitivity to anticipatory goal conflict (*conflict*) are higher-order dimensional dysfunctions in psychopathology that are indexed by neurally and functionally distinct theta components. We also show that there are no clear breaks between functional and dysfunctional theta activity. So, theta-related vulnerability factors to psychiatric illnesses apply to the general population as well as patients with sub-threshold diagnoses.
